# Perceived risks of COVID-19, attitudes towards preventive guidelines and impact of the lockdown on students in Uganda: A cross-sectional study

**DOI:** 10.1371/journal.pone.0266249

**Published:** 2022-04-04

**Authors:** Ngwa Niba Rawlings, Emmanuela Ambe Akwah, James Musisi, Kimonia Awanchiri, Rachel Babirye, Diana Emalieu, Lawrence Nduhukyire, Ronald Kakeeto, Lem Ngongalah

**Affiliations:** 1 Department of Research and Development, The Collaboration for Research Excellence in Africa (CORE Africa), Douala, Cameroon; 2 Department of Health Policy & Management, School of Public Health, Makerere University, Kampala, Uganda; 3 Faculty of Health Sciences, Islamic University, Mbale, Uganda; 4 Department of Epidemiology and Biostatistics, School of Public Health, Makerere University, Kampala, Uganda; Satyawati College (Eve.), University of Delhi, INDIA

## Abstract

**Background:**

This study explored students’ perceptions of COVID-19 risks and preventive measures and assessed the impacts of the national lockdown on students in Uganda.

**Methods:**

A web-based survey was conducted to explore students’ perceived risks of COVID-19 and preventive measures; sources of COVID-19 information and impacts of the lockdown. Both undergraduate and post-graduate students (n = 398) participated in the study. Data were analysed using IBM-SPSS-26.

**Results:**

Students acknowledged COVID-19 as a health risk, and their preventive behaviours were influenced by age, gender, marital status and living situation. Most students followed face mask guidelines but did not comply with lockdown restrictions. Social distancing was not always possible due to overcrowding. Students’ main sources of COVID-19 information were local media (e.g., TV, radio) and social media. Most students (especially females) were unable to access online learning platforms due to poor internet connectivity, high costs and no access to computers. Meanwhile, a majority of those who studied online did not enjoy the experience. Students experienced depression, frustration, stress and anxiety during the lockdown; became less physically active and spent most of their time on social media, sleeping, eating or watching movies. Some students indulged in smoking, drinking alcohol, taking drugs and gambling for their first time, while others did them more often than before.

**Conclusion:**

The increase in sedentary activity, poor mental health and substance use over the lockdown period puts students at risk of health complications and poses a potential threat to the healthcare system. These risks may also negatively impact their future learning and academic potential. Further research is needed to understand the transitional experiences of students between physical and virtual learning, and how they can be supported. There is also a need to ascertain the feasibility of guidelines such as social distancing in developing countries, to increase compliance.

## 1. Introduction

The COVID-19 pandemic has had devastating impacts on education, health, social life and general wellbeing [1–3]. About half of the world’s population (3.3 billion) faced a risk of losing their livelihoods, with millions of people being at risk of falling into extreme poverty [4]. As of March 2021, there had been 117,132,788 confirmed cases of COVID-19 globally, and 2,600,839 deaths from 223 countries, according to the World Health Organization (WHO) [5]. In Africa, there were over 2,909,543 confirmed cases in 12 countries, with more than 73,723 deaths [5]. Estimates for Uganda stood at 40,485 cases and 334 deaths by March 2021 [5].

The WHO and other health agencies regularly provide information on how to prevent, manage and mitigate the effects of COVID-19, to help reduce the impact of the pandemic [6]. Several countries including Uganda implemented national lockdowns, as a way of controlling the spread of the virus, by reducing physical contact between people [7]. Lockdowns are useful for preventing mass casualties from the emergence of a new virus in an immunologically naïve population, especially in the absence of effective therapies or vaccines [8]. However, lockdowns also have negative impacts (e.g. health, economic, social, psychological and academic impacts) [8,9] which could be difficult to quantify, and may be overlooked.

The national lockdown in Uganda involved closures of educational institutions, restrictions on public gatherings, limitations on public transportation and a nation-wide curfew from 7pm to 6.30 am [7]. Due to the halt in education and suspension of face‐to‐face lectures, most students around the world have had to continue pursuing their studies online [2]. However, this is particularly challenging for students in developing countries such as Uganda, where internet connectivity is poor and access limited [7]. According to the United Nations Development Programme (UNDP), more than 17.5 million students in both public and private schools in Uganda were at home due to the closure of schools, and were unable to access educational programmes delivered online [7]. The lockdown also led to job losses and reduced income-generating opportunities, especially for people working in the informal sector, most of whom are youths [7]. School closures have also kept students idle at home, with reports showing an increase in unwanted pregnancies, negative social behaviours and adverse psychological outcomes [10,11].

While nation-wide interventions such as lockdowns are usually made mandatory to the public, the success of such interventions in controlling the spread of a disease is largely dependent on the public’s understanding of the risks of the disease and response to preventive measures. Knowledge gaps on COVID-19 preventive measures have been reported among various groups of people in Uganda, including students, healthcare workers and people working in the informal sector (e.g., drivers and security personnel) [12–14]. To our knowledge, there are limited studies exploring students’ perceptions of the risks of COVID-19 and their attitudes towards the preventive measures adopted in Uganda. There are also limited studies exploring the impacts of the lockdown on student’s health and social wellbeing. This study aimed to explore students’ perceptions of COVID-19 risks and preventive measures, and to assess the health, social and financial impacts of the national lockdown measures on students in Uganda.

## 2. Materials and methods

A web-based cross-sectional survey was used to collect data from randomly selected students in Uganda. The survey questionnaire was developed using the John Hopkins University COVID-19 Community Response Survey Guidance [15], and covered three areas: (i) students’ sociodemographic characteristics, (ii) perceived risks of COVID-19 and attitudes towards preventive guidelines (face mask recommendations, lock down restrictions and social distancing); (iii) students’ sources of COVID-19 information; and (iv) impacts of the lockdown on students.

Eligible participants for the study were students at tertiary level of education (Universities and other higher education institutions). Surveys were distributed online through emails, social media platforms (WhatsApp, Facebook, twitter) and CORE Africa’s student networks in Uganda. Online surveys have been identified as a useful means of assessing knowledge and perceptions among the general population, especially during fast-moving infectious disease outbreaks [16]. Sample size calculations were done using the Kish-Lislie (1965) survey sampling technic, yielding a target of ≥384 students for a representative sample of the student population in Uganda. The survey was piloted with 10 randomly selected Ugandan students to assess its readability and validity, before final distribution. Data collection was done from June to September 2020. The study was conducted following the Checklist for Reporting Results of Internet E-Surveys (CHERRIES) guidelines [17].

### 2.1 Ethical considerations

This study was guided by the Helsinki Declaration as revised in 2013. The study purpose was briefly explained on the study questionnaire and participants were required to give their consent (including parental/guardian consent for those younger than 18 years) before filling the questionnaire. No personally identifiable information such as names or contact information were collected during the study. All participant data were maintained in strict confidentiality. Participants were informed that their participation was completely voluntary, and that there was no compensation for taking part in the study. The study was approved by the CORE Africa ethics committee (Ref:1007/0464/2020).

### 2.2 Data analysis

The collected data were coded and validated in duplicate. Data analysis was done using IBM SPSS version 26. Frequencies and proportions were calculated for participants’ sociodemographic characteristics (age, gender, level of education, living situation, marital status, religion, and number of children) and chi-square tests were used to determine associations between these characteristics and the students’ perceived risks and behaviours. A p-value of less than 0.05 was considered statistically significant. Where significant associations were identified, sub-group post-hoc analyses were performed using Bonferonni adjusted residuals [18].

## 3. Results

### 3.1 Sociodemographic characteristics

Three hundred and ninety-eight (398) students took part in this study– 51.3% male and 48.7% female (Table 1). Participants’ ages ranged from 16 to 42, with a majority being between 16–25 years (48.7%). Most students were undergraduates (66.8%), single (77.4%), living with family or friends (69.1%) and Christian (87.9%). About a third (34.7%) had children.

**Table 1:** Showing Sociodemographic characteristics of study participants.

Characteristic	n	%
**Age group** 16–25 26–30 31–35 35+	194 114 66 24	48.7 28.6 16.6 6.0
**Sex** Male Female	204 194	51.3 48.7
**Level of education** Undergraduate Postgraduate	266 132	66.8 33.2
**Marital status** Married Single	90 308	22.6 77.4
**Has children** Yes No	138 260	34.7 65.3
**Living situation** Alone With family or friends	123 275	30.9 69.1
**Religion** Christian Muslim	350 48	87.9 12.1

### 3.2 Students’ perceived risks of COVID-19, awareness of prevention guidelines and attitudes towards preventive measures

A majority of the students acknowledged that COVID-19 is real (71.6%), is a threat to health (96.2%), and that everyone is at risk of contracting the virus (79.6%) (Table 2). However, some students believed that it was deliberately created or released by people (31.7%), while a few were uncertain about whether it is real or not (28.4%).

**Table 2:** Perceived risks of COVID-19 and attitudes towards preventive guidelines.

	n	%
**Do you think COVID-19 is real?** Yes, I think it is real Yes, I think it is real but it was deliberately created or released by people I am uncertain about whether it is real or not	159 126 113	39.9 31.7 28.4
**What do you think about your risk of getting infected with COVID-19?** I think everyone is at risk of catching the virus I don’t think I’m at risk of catching the virus	317 81	79.6 20.4
**Do you think COVID-19 is a threat to health?** Yes, I think it is a threat to health No, I don’t think it is a threat to health	383 15	96.2 3.77
**Are you aware of the guidelines on how to protect yourself from COVID-19?** Yes No	380 18	95.5 4.5
**Have you been observing the lockdown (e.g., no visiting, no gathering)?** Yes Partially No	196 136 66	49.2 34.2 16.6
**Did you wear a face mask when going out during the lockdown?** Yes No Did not go out during the lockdown	256 118 24	64.3 29.6 6.0
**Have you been following Government guidelines on social distancing?** Yes No	193 205	48.5 51.5

Students were generally aware of the guidelines on how to protect themselves from COVID-19 (95.5%) and about half (49.2%) had complied with lockdown recommendations (e.g., no visiting, no gatherings etc), while others either did so partially (34.2%) or not at all (16.6%) (Table 2). Most students wore face masks when going out during the lockdown (64.3), but a majority did not practice social distancing (51.5%) (Table 2).

**Fig 1:**
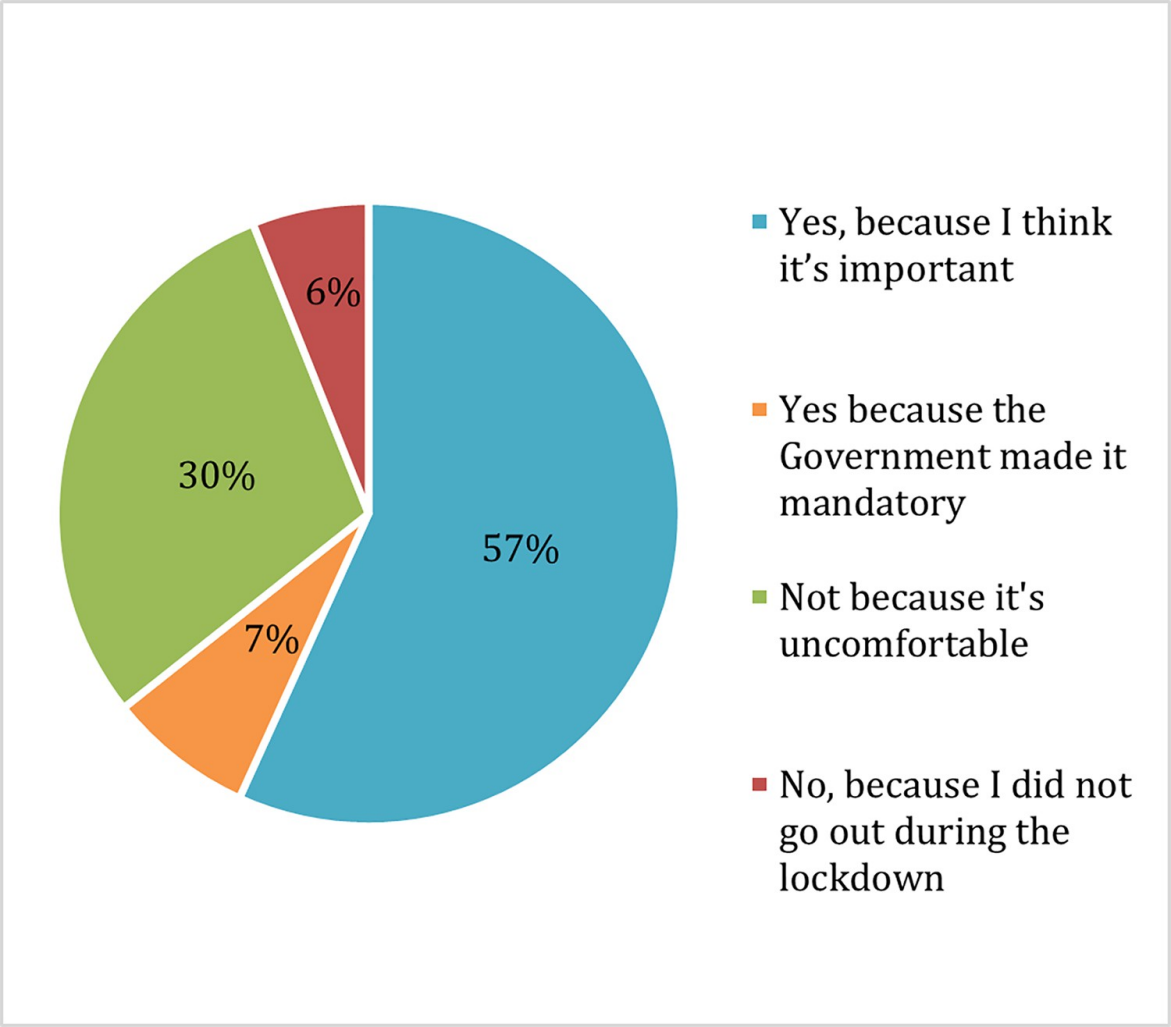
Reasons for wearing and not wearing face masks during the lockdown.

Students gave two main reasons for wearing face masks: because they thought it was important to do so (57%) or because it was mandatory, and they did not want to get in trouble for breaking the law (7%) (Fig 1). Meanwhile, students who did not wear face masks did so because they found it uncomfortable (30%) (Fig 1).

**Fig 2:**
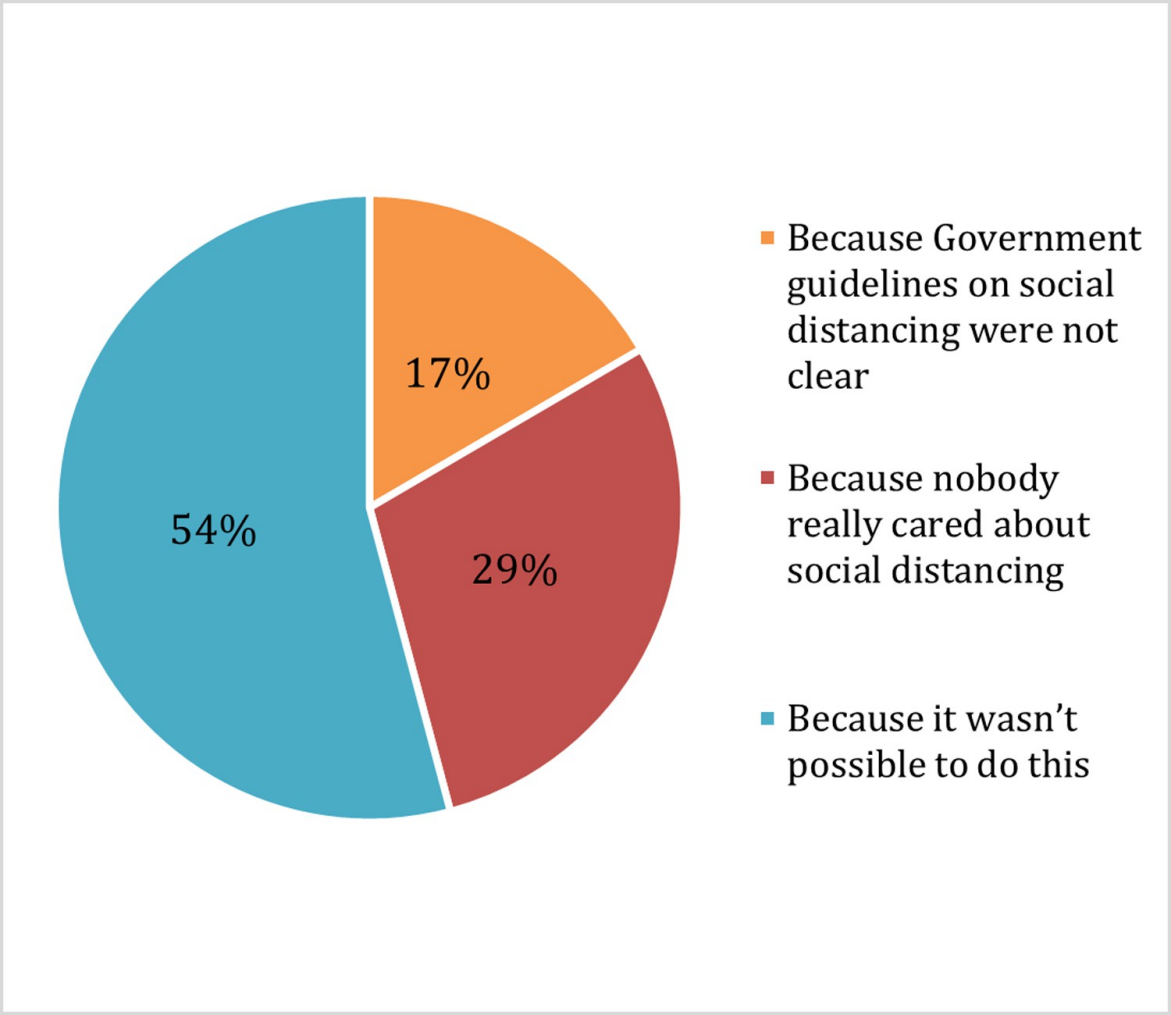
Reasons for not complying with social distancing guidelines.

Students who did not practice social distancing gave three main reasons for this: because it was not feasible to do so due to overcrowding (54%), because social distancing did not appear to be an important issue for people around them (29%), or because Government guidelines on social distancing were not clear (17%) (Fig 2).

### 3.3 Associations between students’ sociodemographic characteristics, COVID-19 perceptions and preventive behaviours

Age, gender, having children and living situation were significantly associated with COVID-19 threat perceptions following Chi^2^ tests (Table 3). Gender was also significantly associated with students’ perceptions on whether COVID-19 is real or not. For preventive behaviours, there was a significant association between gender and students’ compliance with lockdown restrictions, while age and living situation were associated with face mask use. There were no associations between sociodemographic factors and social distancing behaviour.

**Table 3:** Associations between students’ sociodemographic characteristics and COVID-19 perceptions and preventive behaviours.

	X^2^ test (N = 398)					
	Perceived risk of COVID-19			Preventive behaviours		
	COVID-19 perceived as real	COVID-19 perceived as threat to self	COVID-19 perceived as threat to others	Observing lockdown	Wearing a face mask	Practicing social distancing
**Age** X^2^ df	0.506 3	8.125* 3	31.048* 3	3.641 3	9.196* 3	2.061 3
**Gender** X^2^ df	11.011* 1	9.667* 1	23.313* 1	10.904* 1	0.215 1	1.886 1
**Level of education** X^2^ df	1.673 1	0.574 1	0.211 1	0.727 1	0.473 1	0.323 1
**Marital status** X^2^ df	0.423 1	3.534 1	0.450 1	0.006 1	1.057 1	0.029 1
**Has children** X^2^ df	1.267 1	4.287* 1	0.364 1	3.563 1	2.211 1	1.030 1
**Living situation** X^2^ df	1.402 1	14.161* 1	1.483 1	0.116 1	10.216* 1	0.055 1
**Religion** X^2^ df	2.227 1	2.076 1	3.040 1	0.529 1	0.131 1	0.929 1

**Note:** X^2^ –chi square result; df–degree of freedom; *significant *p*-value of <0.05 (factor was significantly associated with perceived risk or preventive behaviour).

Post-hoc analyses showed significantly higher number of students aged over 35, and female students perceiving COVID-19 as not being a threat (p<0.001) (Table 4A). Differences for other age groups were not significant. Being female was also significantly associated with uncertainty about whether COVID-19 is real or not (p = 0.001). The perception of not being at risk of catching the virus was significantly more common among male students and students who lived alone (p = 0.002).

Post-hoc analyses for preventive behaviours showed that more females complied with lockdown restrictions than males (p = 0.001); and students who lived alone were less likely to wear face masks than those who lived with other people (p = 0.001, Table 4B). Age group differences for face mask use were not significant.

**Table 4a:** Post-hoc adjusted residuals for risk perceptions with Bonferroni correction.

Perception of COVID-19 as a threat	Yes	No	p-value
Age (p adj = 0.006) 16–25 26–30 31–35 Over 35	-2.1 3.0 2.1 -4.5	2.1 -3 -2.1 4.5	NS NS NS <0.001*
Gender (p adj = 0.012) Male Female	4.8 -4.8	-4.8 4.8	<0.001*
**Perception of COVID-19 as real**	**Yes**	**Uncertain**	**p-value**
Gender (p adj = 0.012) Male Female	3.3 -3.3	-3.3 3.3	0.001*
**Perception of being at risk of catching the virus**	**Yes**	**No**	**p-value**
Gender (p adj = 0.012) Male Female	-3.1 3.1	3.1 -3.1	0.002*
Living situation (p adj = 0.012) Living alone Living with other people	-3.1 3.1	3.1 -3.1	0.002*

**Note:** p adj–adjusted *p*-value; NS–not significant; *significant *p*-value (statistically significant difference between groups).

**Table 4b:** Post-hoc adjusted residuals for preventive behaviours with Bonferroni correction.

Complied with lockdown restrictions	Yes	No	p-value
Gender (p adj = 0.012) Male Female	-3.3 3.3	3.3 -3.3	0.001*
**Used face mask**	**Yes**	**No**	
Living situation (p adj = 0.012) Living alone Living with other people	-3.2 3.2	3.2 -3.2	0.001*

**Note:** Adj–adjusted *p*-value; *significant *p*-value (statistically significant difference between groups).

### 3.4 Students’ sources of COVID-19 information and perceptions of trusted sources

**Fig 3:**
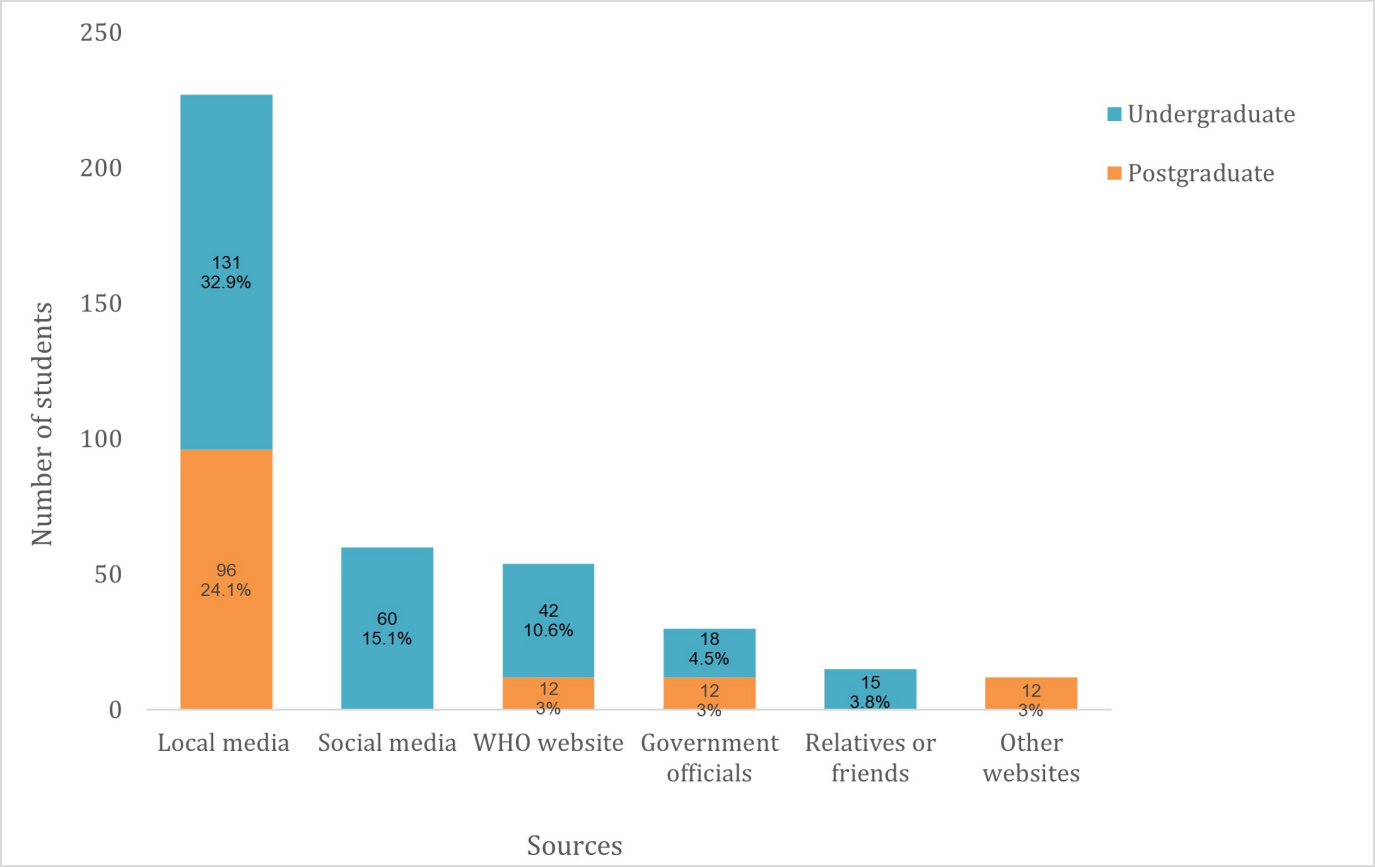
Sources of COVID-19 information.

Students’ main source of information about COVID-19 was local media (such as national TV and radio stations) (57%) followed by social media (15%) and the WHO website (10.6%) (Fig 3). Only undergraduate students reported getting information from social media or from family and friends.

**Fig 4:**
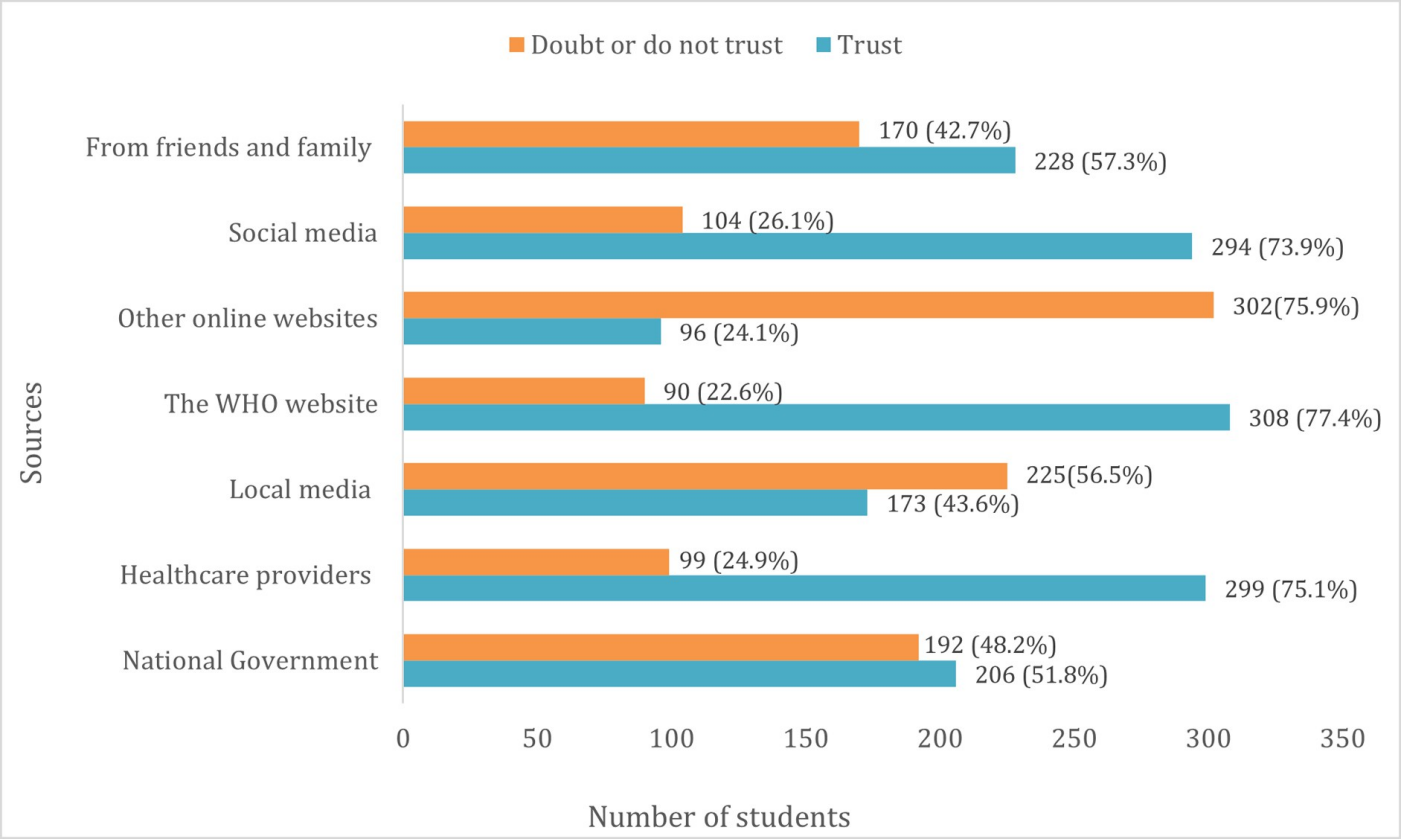
Students’ trust on sources of COVID-19 information.

Although local media was reported as the main source of information, it was not amongst the three sources students trusted most. The WHO website was the most trusted information source (77.4%), followed by health providers (75.1%) and social media (73.9%) (Fig 4).

### 3.5 Impact of the national lockdown due to COVID-19 on students

#### 3.5.1 Impact on students’ education

**Fig 5:**
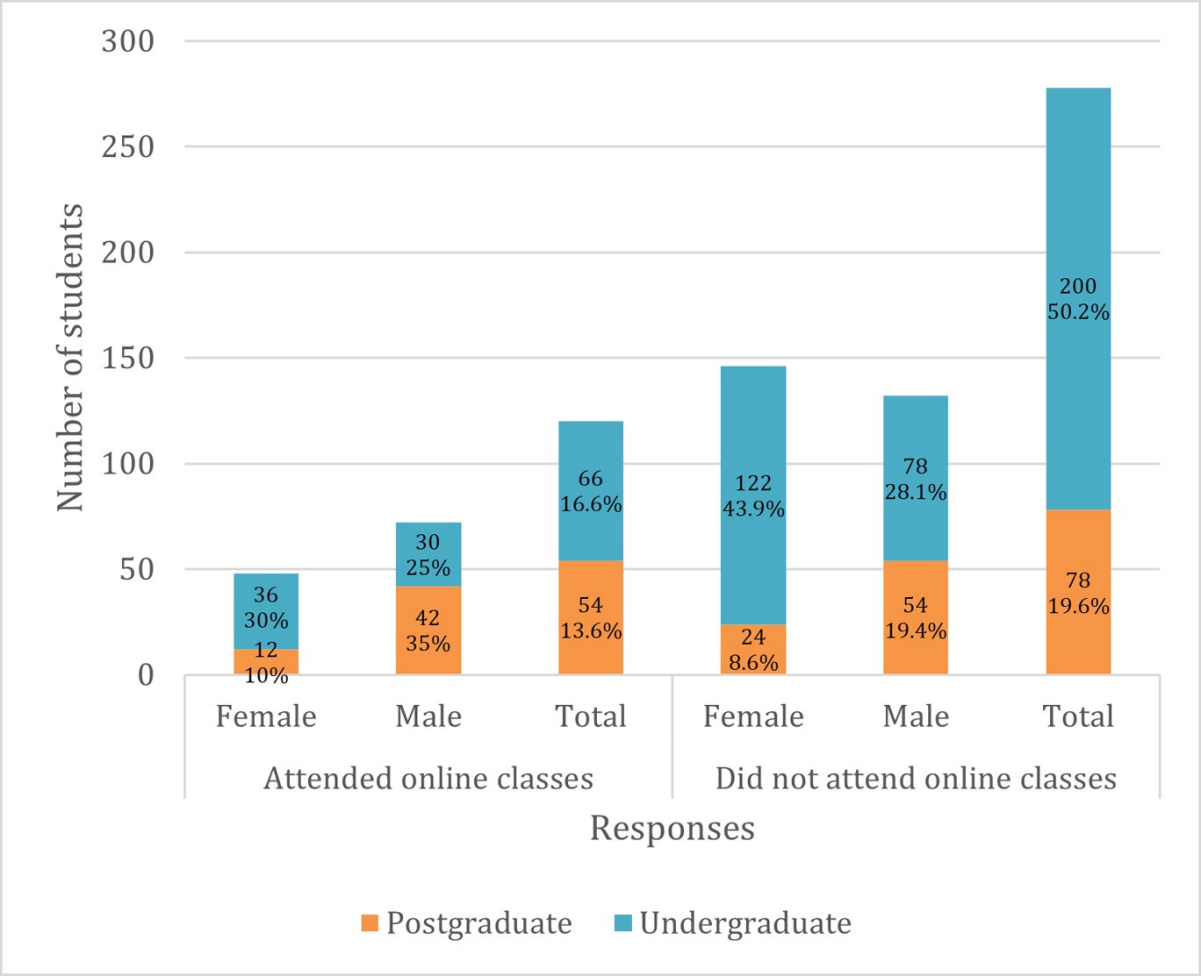
Students’ access to online classes.

**Fig 6:**
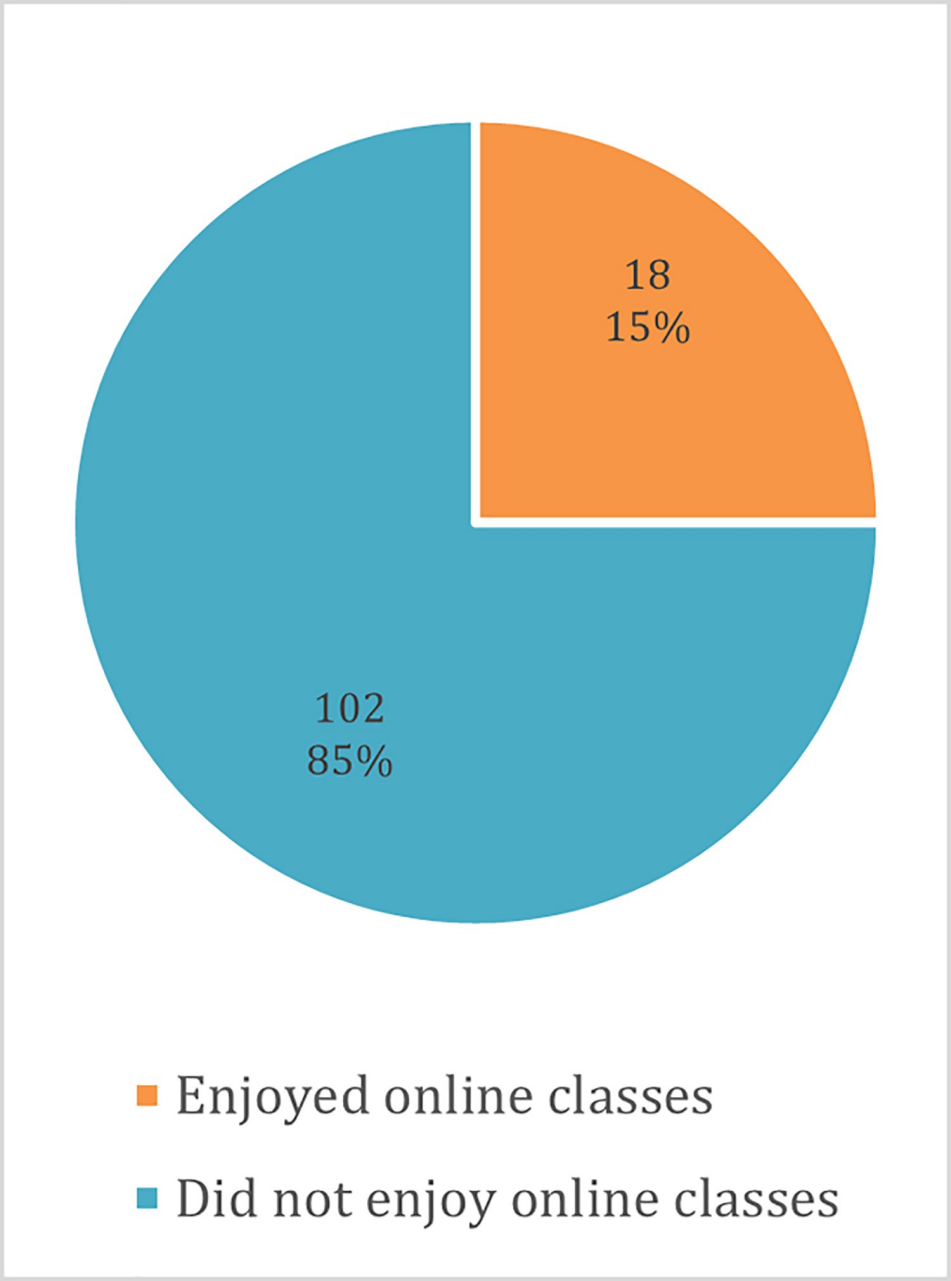
Students’ experiences of attending online classes.

A majority of the students (69.8%) were unable to attend classes online during the lockdown, and there were gender differences among students who were able to attend as well as those who were unable to. More males (60%) than females (40%) continued attending classes online, while more females (52.5%) than males (47.4%) did not attend (Fig 5). Various reasons were reported for students not being able to attend classes online, such as poor internet connectivity, high cost of internet data and lack of computers. Of those who were able to continue attending classes online, a majority (85%) did not enjoy the experience (Fig 6).

#### 3.5.2 Impact on students’ access to and use of health services

**Fig 7:**
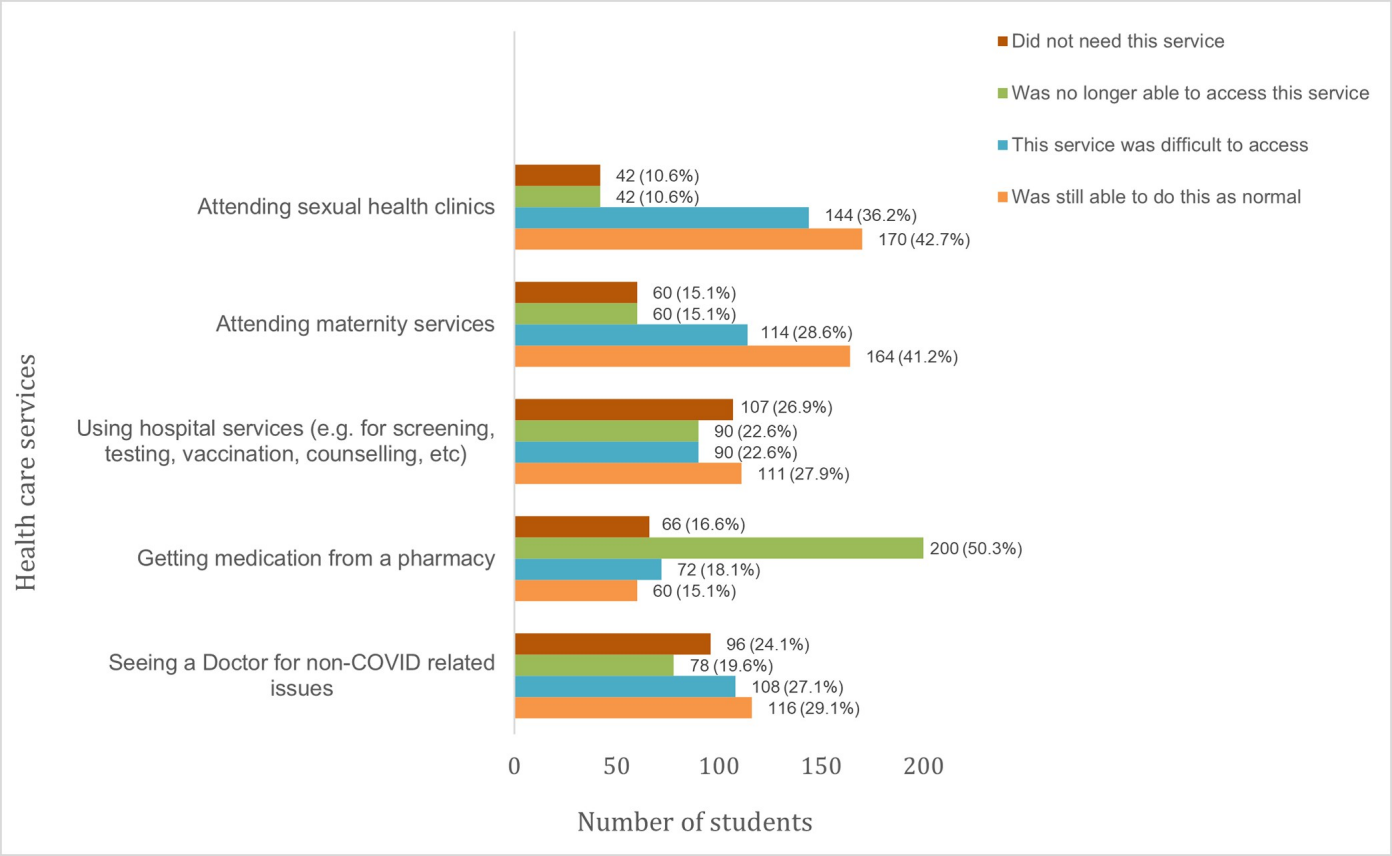
Students’ access to and use of health services during the lockdown.

About a third of students (31.2%) reported that they were able to access health services as normal during the lockdown (Fig 7). Meanwhile, several others either faced difficulties or could not access sexual health clinics (46.8%), maternity services (43.7%), screening and counselling services (45.2%) and routine Doctor consultations (46.7%) (Fig 7). Half the students (50.3%) were also not able to access pharmacies to buy medication.

#### 3.5.3 Impact on students’ mental health

**Fig 8:**
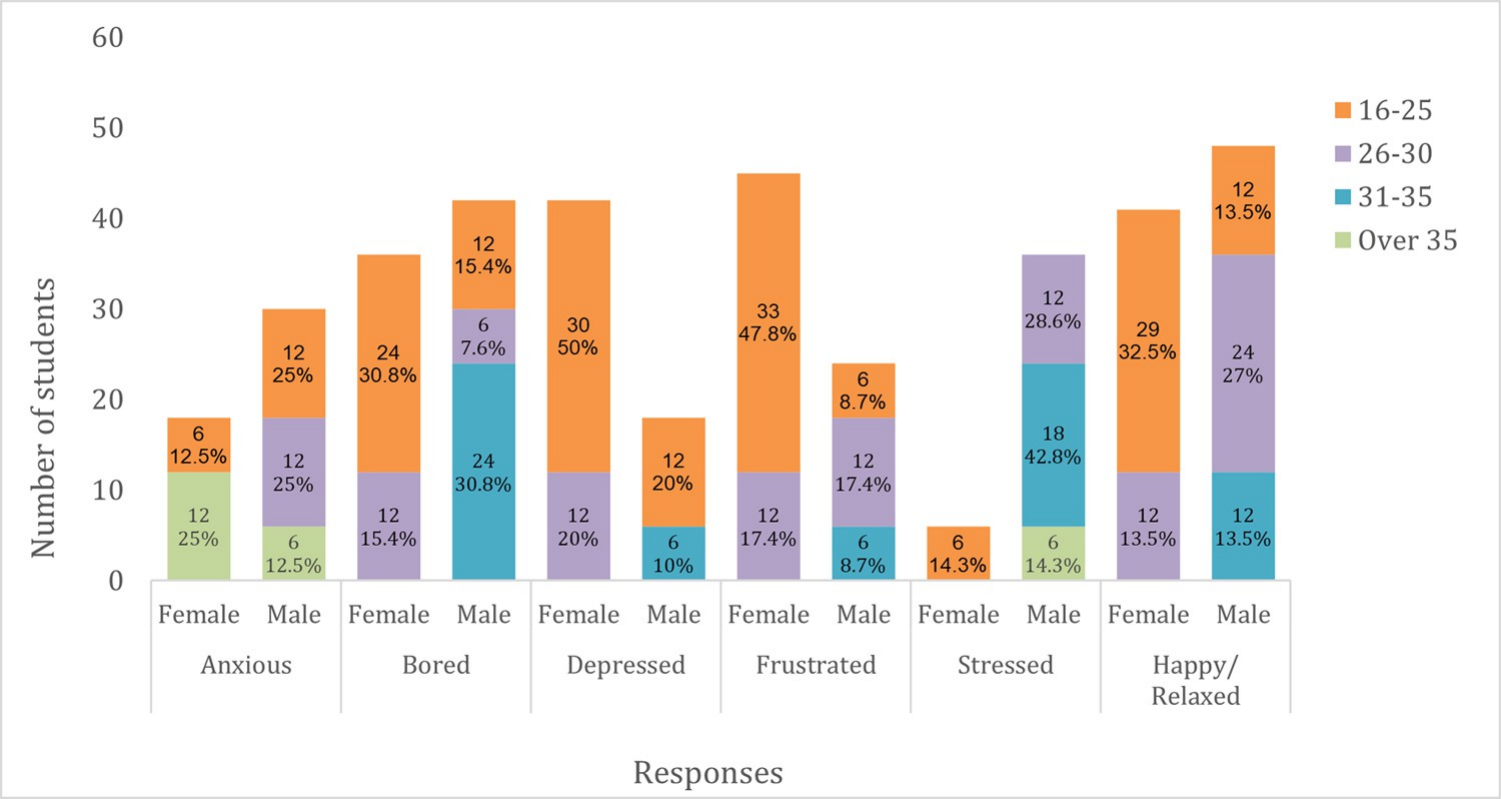
Mental health traits during the lockdown.

A majority of students experienced negative mental health traits during the lockdown, such as anxiety, depression, stress and frustration (Fig 8). Depression, frustration and boredom were commonly reported by young females (aged 16–30), while older females (aged over 35) were more anxious. For male students, stress was commonly reported by those aged 31 and above, while younger males (aged 16–30) were more anxious.

Students also reported feeling happy or relaxed during the lockdown, and this was more common among younger students (aged 16–30)–both male (40.5%) and female (46%). No student over the age of 35 reported feeling happy or relaxed during the lockdown. Positive impacts of the lockdown included having more time to spend with loved ones (39.6%), getting more sleep (15%), having more time to study (9%), and having more time to care for self and family (1.5%).

#### 3.5.4 Impact on students’ social life and daily activity

**Fig 9:**
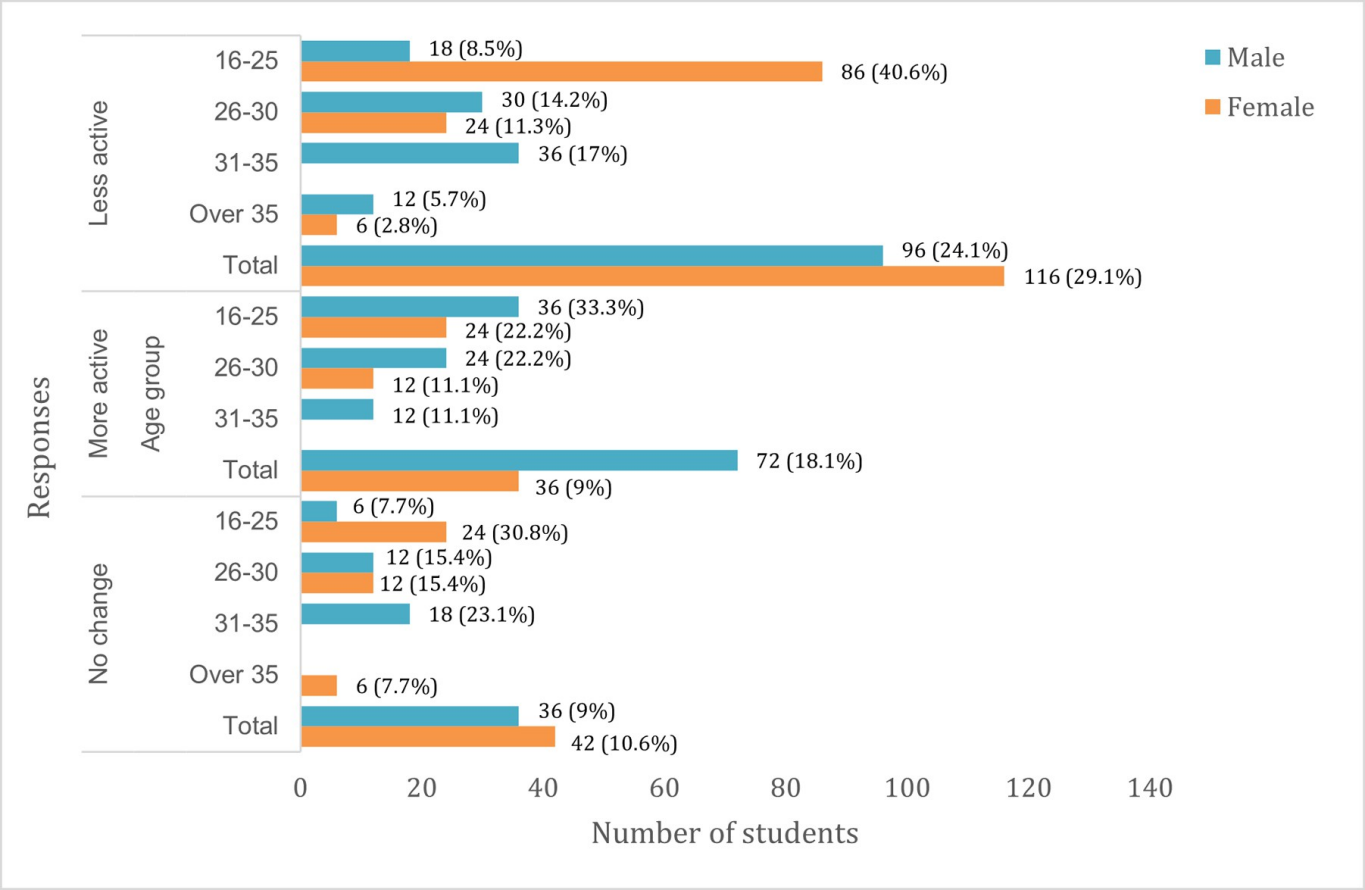
Student’s physical activity level.

**Fig 10:**
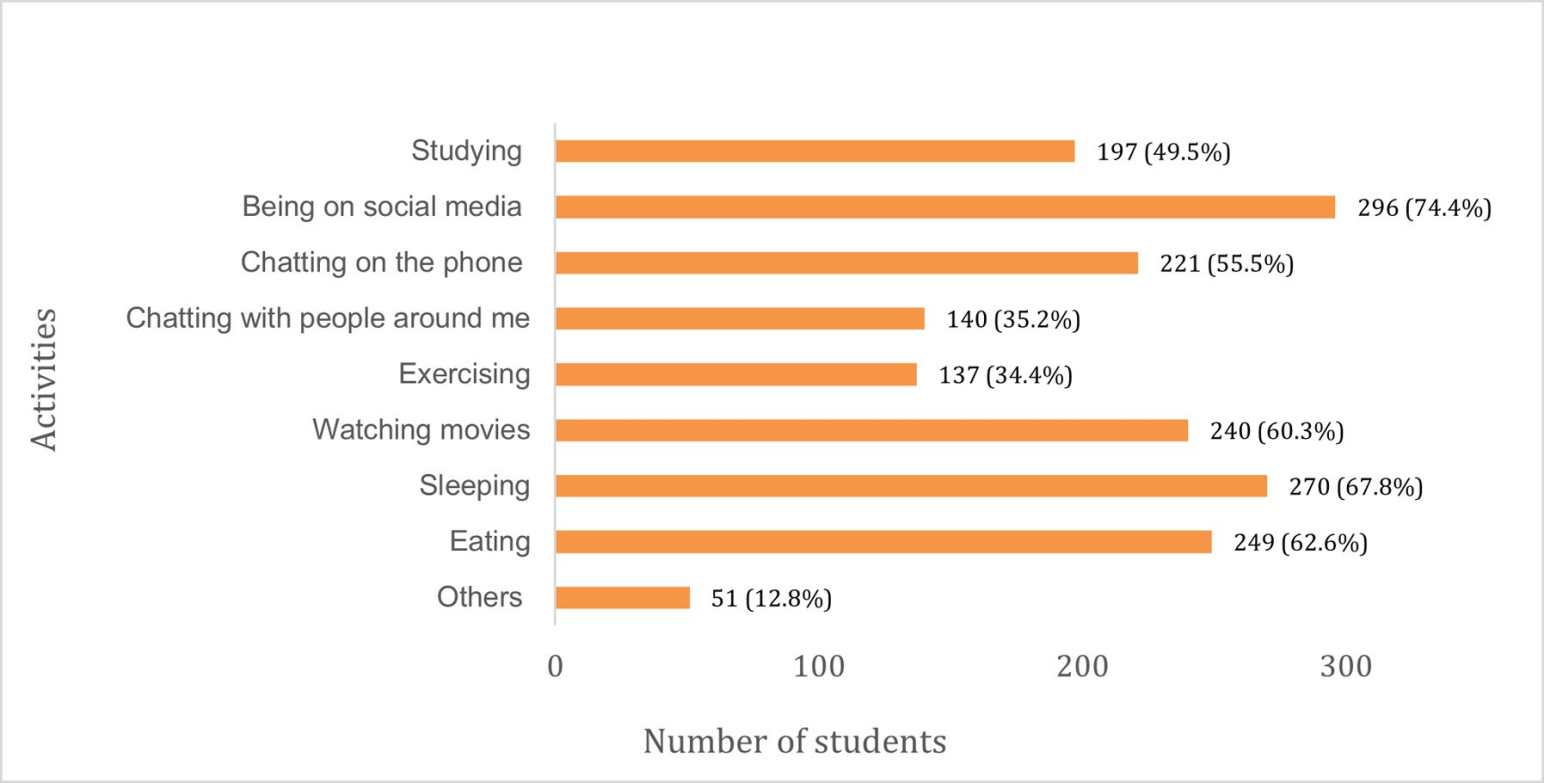
How students spent most of their time during the lockdown.

Over half the students (53.2%) became less active during the lockdown, and physical activity levels were most decreased for females aged 16–25 years (Fig 9). In response to what they spent most of their time doing during the lockdown, students mostly reported being on social media (74.4%), sleeping (67.8%), eating (62.6%), watching movies (60.3%) and talking on the phone (55.5%) (Fig 10).

**Fig 11:**
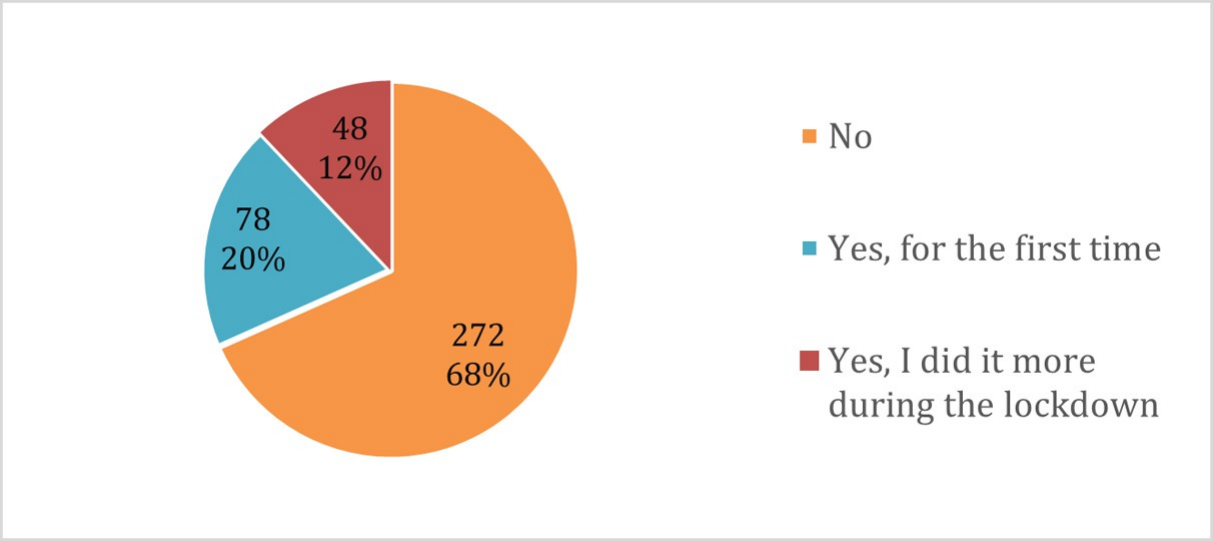
Involvement in substance use and gambling during the lockdown.

The questionnaire also assessed for substance use (smoking, drinking alcohol and taking drugs) and engagement in gambling activities during the lockdown (Fig 11). Although a majority of the students (68%) did not engage in any of these activities, some students reported having been involved in smoking, drinking alcohol, taking drugs or gambling for their first-time during lockdown (20%), while those who used to do these before the lockdown reported doing them more often during the lockdown (12%).

Age and gender were significantly associated with alcohol use, drug use and gambling; but not smoking (Table 5). Post-hoc analyses showed that more male students started drinking alcohol and gambling for the first time than females (adjusted residual *p* = 0.04), while taking up alcohol and gambling for the first time were most common among students aged 26–30 (adjusted residual p<0.001).

**Table 5:** Associations between students’ sociodemographic characteristics, substance use and gambling.

X^2^ test (N = 398)				
	Alcohol use	Smoking	Drug use	Gambling
**Age** X^2^ df	72.19* 6	7.13 3	32.50* 6	63.01* 6
**Gender** X^2^ df	12.33* 2	0.008 1	8.74* 2	20.17* 2

**Note:** X^2^ –chi square result; df–degree of freedom; *significant *p*-value of <0.05 (factor was significantly associated with perceived risk or preventive behaviour).

#### 3.5.5 Impact on students’ finances

A majority (72.4%) of the students reported feeling generally worried about their financial situation both during and after the lockdown. Although there was no change in employment status for most students during lockdown (60.8%), some students lost their jobs (18.1%) and others were worried that they may not be able to get their jobs again after the lockdown (16.6%).

## 4. Discussion

This study explored students’ perceptions of COVID-19 risks, their sources of information on COVID-19 and their attitudes towards the nation-wide preventive measures adopted in Uganda. It also assessed the financial, health and psychosocial impacts of the lockdown on students. Students generally acknowledged COVID-19 as being a threat to human health and were aware of recommended guidelines for COVID-19 prevention. While most students complied with face mask recommendations, more than half did not follow lockdown restrictions. Similar trends have been noted in countries like South Africa, where students threw parties, ignoring lockdown rules [19]. In addition to public health guidelines, younger populations may also benefit from tailored messages addressing the consequences of their non-compliance and individual actions on the community, especially since they are more used to life out of home.

The findings from this study also reveal concerns about the feasibility of social distancing in an African context, as more than half the students were unable to practice social distancing. Reports have been made in various African countries about people being unable to comply with social distancing measures due to extended households, informal settlements, political unrest and cultural/religious activities [20,21]. Other authors have noted factors like disorganised and congested markets with large numbers of traders and consumers within a confined location; unlike in developed countries where markets and shopping malls are more organised, making it easier for people to follow social distancing measures [22]. Cultural and religious events such as naming ceremonies, church gatherings, weddings and funerals have also been shown to undermine the success of national lockdowns in African countries [23].

Students’ perceptions of COVID-19 risks and attitudes towards preventive measures were significantly associated with age, gender, level of education, marital status and living situation, but not religion. These findings are consistent with those of other studies [24,25]. Knowledge of COVID-19 has been shown to influence perceptions of its risks, and subsequently the adoption of preventative behaviours [24]. Conspiracy theories such as the idea that COVID-19 was intentionally created as was reported by some students in this study, have also been identified in other African countries [20]. Such ideas could lead to an under-estimation of the risks and influence negative attitudes towards preventive recommendations.

Students faced challenges accessing learning platforms online due to poor internet connectivity, expensive internet data packages and lack of access to computers. Connectivity issues have also been reported as a main barrier to the implementation of e-learning in universities in Uganda during COVID-19 [26]. A higher proportion of female students were unable to study online compared to males, which exacerbates the gender gaps in Africa, where gender inequalities in education already prevail [27]. Online learning is a useful way of advancing students’ digital skills and has the potential to enhance their performance and learning outcomes [28,29]. However, these benefits will only be reaped if students are able to access learning platforms online.

Although some students were able to study online, a majority did not enjoy the experience, which may suggest that these programmes were not meeting the needs of the students. Besides challenges relating to cost and internet access, some authors have reported stress among students due to the complexities of online education [30]; and a lack of locally developed course content that reflects the context of African countries, as most e-learning courses are designed following foreign learning material [31]. Further research with both students and learning institutions would be useful in exploring ways of maximising the potential of e-learning and improving the experience for African students.

The lockdown had negative psychological impacts on students, causing them to experience depression, frustration, stress and anxiety. These may partly be attributed to the challenges they were experiencing, such as not being able to study, job losses, difficulties in accessing essential health care services and fear of the unknown. These psychological effects could be detrimental to both the health and learning capacity of students in the long term [32–35]. There were also trends of decreased physical activity and increased sedentary behaviours during the lockdown; and students getting involved in smoking, drinking alcohol and taking drugs; which further heightens their susceptibility to poor health [36].

The findings from this study are novel and make several important contributions to the literature. Identifying differences in behaviour by age, gender and other sociodemographic factors contributes to the understanding of patterns in the public’s responses to national guidelines and are useful to consider when communicating public health messages with young populations. Some participants in this study, though a minority, also felt that Government guidelines on social distancing were unclear. This study also identifies a need to ascertain the feasibility of public health guidelines such as social distancing in local African contexts, in order to increase compliance.

The health risks shown in this study have important implications for the healthcare system in Uganda, as the culmination of these risks over the lockdown period could lead to an increased demand for healthcare services in the long term. This emphasises a need to think beyond the immediate impacts of COVID-19, especially in developing countries where health systems are fragile and under-resourced [37].

Our findings are limited as the data were collected through online sources, and students who did not have access to the internet were not captured in the sample. Our results may thus not be representative of that population. Further research would be useful in understanding the behaviours and impacts of the lockdown on these students. Secondly, qualitative studies would be useful in further exploring the lived experiences of students, especially relating to their transitional experiences between physical and virtual learning and how they can be supported.

## 5. Conclusion

This study showed associations between students’ sociodemographic characteristics and their attitudes towards COVID-19 prevention guidelines. While most students complied with face mask recommendations, more than half did not follow lockdown restrictions and social distancing was not always feasible mainly due to overcrowding. Sedentary activities increased during the lockdown and students experienced depression, frustration, stress, and anxiety. Several students indulged in drinking alcohol, smoking, taking drugs and gambling. The increase in sedentary activity, substance use and poor mental health over the lockdown period increases students’ vulnerability to health complications and may also have negative consequences on their subsequent learning and performance. Further research would be useful in exploring the lived experiences of students during the lockdown and in identifying strategies to support their learning and development.

## Supporting information

S1 Table Study data. (XLSX)
